# A Cross-Sectional Study of Determinants of Type 2 Diabetes Mellitus Among Professional Drivers in the Perambalur Municipality Area of Tamil Nadu, India

**DOI:** 10.7759/cureus.34321

**Published:** 2023-01-29

**Authors:** Tamilarasan M, Karthikeyan Kulothungan, Shagirunisha Rizvana, Sriranganathan Thirunavukkarasu

**Affiliations:** 1 Community Medicine, Dhanalakshmi Srinivasan Medical College and Hospital, Perambalur, IND; 2 Community Medicine, Srinivasan Medical College and Hospital, Tiruchirappalli, IND

**Keywords:** driving, risk factors, professional drivers, diabetes mellitus, obesity, hypertension

## Abstract

Background

Professional drivers have a powerful impact on public safety. They are also at a higher risk of obesity, hypertension, and type 2 diabetes mellitus (T2DM) because of their lifestyle. Diabetes and its complications can affect driving and cause increased road traffic accidents. This study aimed to estimate the prevalence of T2DM and determine the risk factors contributing to the development of T2DM among professional drivers in the Perambalur Municipality of Tamil Nadu, India.

Methodology

This cross-sectional study was carried out between September 2022 and December 2022 among 118 private bus drivers and full-time, professional, three-wheeler drivers in the Perambalur Municipality. A pre-tested semi-structured proforma was used to collect information on the driver's socio-demographic profile and to inquire about their diabetes history, which was verified with their records. We elicited the risk factors of T2DM among those drivers. We recorded the anthropometric measurements and blood pressure. Data analysis was done using IBM SPSS Statistics for Windows, Version 21.0 (Released 2012; IBM Corp., Armonk, New York, United States).

Results

Out of 118 study participants, the majority were in the age group of 51-65 (37.3%). Seventy-seven of the participants have completed their secondary education, and 38 of them belong to the class 2 socioeconomic class. Three-fourths of the sample (83.1%) belonged to nuclear families. Around one-third were current smokers, one-fourth had the habit of chewing tobacco, and more than half of the participants consumed alcohol. Nearly 83.7% had moderate physical activity, followed by 11.9% who had heavy activity, and 5.1% who did not do any physical activity. The prevalence of T2DM among professional drivers was 11.9%. The risk factors that contributed to the development of T2DM among professional drivers were age, education, smoking, tobacco chewing, hypertension, elevated BMI, and elevated WC, which are statistically significant (p˂0.05).

Conclusion

We found the proportion of obesity, hypertension, and diabetes to be higher among professional drivers than among the general population. This demands an urgent need for preventive and health-promotive interventions to address these chronic diseases.

## Introduction

Drivers are a part of professional groups whose activities have a strong impact on public safety [1]. Since driving is a sedentary occupation with changing day-night shifts affecting their circadian rhythm, professional drivers are at higher risk for developing obesity, hypertension, type 2 diabetes mellitus (T2DM), and cardiovascular diseases in later stages of life [2].

Diabetes mellitus is caused by a complex interaction of genetic and environmental factors [3]. Moreover, the stress of driving and exposure to atmospheric pollutants as risks can influence their performance, and cause sickness and absenteeism, thereby posing a great financial burden to society [4].

Complications of T2DM like diabetic retinopathy and neuropathy can affect driving skills. Diabetic neuropathy can cause muscle weakness, foot ulcers, and even lower extremity amputations [5]. Irregular treatment and skipping medications can result in hyper/hypoglycemia and may lead to increased reaction time, imbalance, and loss of consciousness. Such health issues that affect drivers may result in an increased risk of road accidents [6].

Noncommunicable diseases (NCD) are the major challenges to sustainable development in the 21st century. Goal 3 of Sustainable Developmental Goals (SDG), thus, focuses on reducing premature mortality due to NCDs by at least one-third and providing access to affordable medicines for NCDs [7]. To the extent of our knowledge, very few studies have been done in India to assess other comorbidities like cardiovascular morbidity profile [8].

Many studies done in other parts of the world have reported that professional truck drivers and other road transport professionals have a higher risk of ischemic heart disease and metabolic syndrome [9]. The studies conducted in India showed that the risk factor for the development of T2DM was higher among professional drivers who were chronic smokers, used chewable tobacco products, and addicted to alcohol [10].

In contrast to the above findings, a cross-sectional study conducted in June 2011 among 59 truck drivers in India by Sharma et al. found that the prevalence of risk factors for metabolic disorders was lower among long-haul truck drivers than in the general population [11]. So, we aimed to conduct a study to estimate the prevalence of T2DM and to determine the risk factors contributing to the development of T2DM among professional drivers in Perambalur Municipality of Tamil Nadu, India.

## Materials and methods

Study design and setting

This cross-sectional study was conducted among occupational male drivers in the Perambalur Municipality, Tamil Nadu, India, from September 2022 to December 2022. Tamil Nadu (formerly Madras State) is situated in the southeast part of India, with Chennai (formerly Madras city and the southern headquarters of British India) as the state capital. Perambalur is an inland district about 200 miles south that is rich in culture, fortresses, and places of worship.

Ethical clearance and informed consent

Before the study began, we got an ethical clearance certificate from the Institutional Ethics Committee (IEC) of Dhanalakshmi Srinivasan Medical College and Hospital, Perambalur, Tamil Nadu, India (Approval number: IECHS/ IRCHS/ N0: 206 B dated August 9, 2022). All participants were provided with information about the study goals before giving consent.

Inclusion and exclusion criteria

All full-time professional drivers with a history of driving for the past one year as their primary job were included. Drivers who are unavailable even after two visits, part-time and occasional drivers, and drivers who had been diagnosed with type 1 diabetes were excluded.

Sample Size

Considering previous data showing that the prevalence of diabetes in India was 7.9% [9], with a 95% confidence level and 5% allowable error, we estimated the sample size to be 112 with a 5% non-response rate. The final estimated sample size was 118. We estimated the sample size using the formula n= Z_1-α/2_^2^PQ/d^2^ (Zα=1.96, P=7.9, Q=92.1, d=5).

Sampling technique

We have selected 118 samples selected by convenience sampling in Perambalur Municiplity. Three-wheeler drivers available at the three-wheeler stands during the visit were taken up for the study.

Study tool

After getting the IEC approval and informed consent from the participants, a pre-tested, semi-structured questionnaire was used to collect socio-demographic data from the drivers including age, caste, religion, education, and income, and we elicited the risk factors of T2DM like lifestyle, dietary habits, habits of alcohol, tobacco chewing, and smoking with the frequency and duration. We enquired about family history and treatment history to identify the risk factors.

Measurements

Height

Height was measured with a stadiometer mounted on a weighing scale to the nearest 0.5 cm. Subjects stood upright without shoes with their back and heads against the height rod, heels together, and eyes directed forward [12]

Weight

For weight measurement, participants stood barefoot on a standardized weighing scale, and weight was measured in kilograms. We asked subjects to wear light clothing, and we recorded weight to the nearest 1 kg [12].

BMI

BMI was calculated using the formula, BMI = weight (kg)/ height (m^2^). Table 1 describes the Asian criteria-based classification of BMI for adults [13].

**Table 1:** Asian criteria-based classification of BMI for adults BMI - Body Mass Index

Body Mass Index (Kg/m^2^)	Grade
Below 18.5	Underweight
18.5-22.9	Normal weight
23-27.5	Overweight
>27.5	Obese

Waist Circumference

The waist was measured using a non-stretchable measuring tape. The participants were asked to stand erect in a relaxed position with both feet together; one layer of clothing was accepted. Waist circumference was measured at a level halfway between the costal margin and iliac crest at the level of the umbilicus, at minimal respiration, measured in a horizontal plane to the nearest 1 mm. In males, waist circumference > 90 cm was classified as obese. The average of three readings was considered the final reading [14].

Blood Pressure (BP)

BP was recorded using a mercury sphygmomanometer following the auscultatory method. Palpated radial pulse obliteration pressure was used to estimate the systolic BP (SBP). We inflated the cuff 20-30 mm Hg above this level for the auscultatory determinations; cuff deflation rate of 2 mm Hg per second was used. Phase I of the Korotkoff sound is the point at which the first sound heard and was used to define SBP, and phase V of the Korotkoff sound is the point at which the sound disappears, and was used to identify diastolic BP (DBP). While measuring BP, the arm ws positioned at heart level while resting on an armrest, and the patient or provider was not talking. Patients were not have consumed stimulants (including smoking) well before the test [8].

We defined hypertension based on the seventh report of the Joint National Committee of Hypertension (JNC 7), which provides a classification of BP for adults aged 18 years or older. In this, hypertensive is defined as a person having a SBP ≥ 140 mm Hg or DBP ≥ 90 mm Hg. A new category designated pre-hypertensive indicates individuals who are at increased risk for progression to hypertension [15]. Table 2 describes the classification of BP for adults [15].

**Table 2:** Classification of blood pressure for adults SBP: systolic blood pressure; DBP: diastolic blood pressure; JNC 7: seventh report of the Joint National Committee of Hypertension

JNC 7 Category	Blood Pressure Reading (SBP/DBP)
Normotensive	< 120 mm Hg / < 80 mm Hg
Pre-hypertensive	120-139 mm Hg / 80-89 mm Hg
Hypertension Stage I	140-159 mm Hg / 90-99 mm Hg
Hypertension Stage II	> 160 mm Hg / >100 mm Hg

Socioeconomic Status

Socioeconomic status was based on Modified BG Prasad’s Classification, updated in 2020 [16]. Table 3 describes the classification of socioeconomic status.

**Table 3:** Classification of socioeconomic status

Social Class	Amount in Rupees/month (per capita monthly income limits)
I	> 7008
II	3504-7007
III	2102-3503
IV	1051-2101
V	< 1050

Statistical analysis

All collected data were entered into Microsoft Excel (Microsoft Corporation, Redmond, Washington, United States), and IBM SPSS Statistics for Windows, Version 21.0 (Released 2012; IBM Corp., Armonk, New York, United States) was used to analyze the results. Frequency and percentage are used to present qualitative data. We also examined the relationship between the risk factors and T2DM using the Chi-square test or Fisher’s exact test, whichever is applicable. We considered a p-value of less than 0.05 statistically significant.

## Results

A total of 118 participants took part in this study. Out of 118, the majority were in the age group of 51-65 years (37.3%) followed by the age group of 36-50 (29.7%). Most of the participants (n=77, 65.3%) completed their secondary education and belonged to class 2 socio-economic class (n=38, 32.2%). Three-fourths of the participants (83.1%) belonged to nuclear families. The complete socio-demographic profile of the participants is given in Table 4.

**Table 4:** Distribution of socio-demographic profile of the study participants

Characteristics		n (%)
Age	21-35	33 (28.0%)
	36-50	35 (29.7%)
	51-65	44 (37.3%)
	> 65	6 (5.1%)
Education	Degree	18 (15.3%)
	Higher Secondary	4 (3.4%)
	Secondary education	77 (65.3%)
	Primary education	13 (11.0%)
	No education	6 (5.1%)
Type of Family	Joint	6 (5.1%)
	Nuclear	98 (83.1%)
	Three generations	14 (11.9%)
Socio- economic status	Class 1	18 (15.3%)
	Class 2	38 (32.2%)
	Class 3	33 (28.0%)
	Class 4	29 (24.6%)

The distribution of personal habits among the participants is given in Table 5. Around one-third were current smokers and one-fourth had a habit of chewing tobacco. More than half of the participants consumed alcohol. The majority (n=98, 83.1%) did moderate physical activity.

**Table 5:** Distribution of personal habits among professional drivers *Tobacco consumption (both smoking and smokeless tobacco), alcohol consumption was classified as follows: Tobacco consumption [17] (a) Smoking Tobacco: · Non-smoker: An adult who has never smoked, or who has smoked less than 100 cigarettes in his or her lifetime. · Ex-smoker: An adult who has smoked at least 100 cigarettes in his or her lifetime but who had quit smoking at the time of the interview. · Current Smoker: An adult who has smoked 100 cigarettes in his or her lifetime and who currently smokes cigarettes, b) Smokeless Tobacco (chewing tobacco, snuff inhalation, Gutka, etc.): · Non-user: A person who has never used smokeless tobacco. · Ex-user: A person who has used smokeless tobacco for one year and not using it at present. · Current user: A person who is using smokeless tobacco while the interview was carried out. **Alcohol consumption was classified as follows: (a) Non-alcoholic: A person who has never consumed alcohol. (b) Ex-alcoholic: A person who consumed alcohol before one year and not consuming at present. (c) Current alcohol consumed: A person who is consuming alcohol at present. ***Physical activity was classified as follows [18]: (a) Sedentary Activity: Person sitting at least 8 hours per day. (b) Moderate Activity: Moderate-intensity sports, fitness or recreational activities that cause a small increase in breathing or heart rate such as brisk walking, cycling, swimming, and volleyball for ≥ 30 minutes per day on ≥ 5 days a week. (c) Rigorous Activity: Vigorous-intensity sports, fitness, or recreational activities that cause large increases in breathing or heart rate such as running and football for ≥ 20 minutes per day on ≥ 3 days a week.

Characteristic		n (%)
Smoking*	Current	44 (37.3%)
	Ex user	16 (13.6%)
	Non-user	58 (49.2%)
Tobacco chewing*	Current	30 (25.4%)
	Ex user	2 (1.7%)
	Non-user	86 (72.9%)
Alcohol consumption**	Current	68 (57.6%)
	Ex user	20 (16.9%)
	Non-user	30 (25.4%)
Diet intake	Mixed diet	110 (93.2%)
	Vegetarian	8 (6.8%)
Additional intake of salt while eating	Yes	34 (28.8%)
	No	84 (71.2%)
Frequent fried food consumption	Yes	40 (33.9%)
	No	78 (66.1%)
Predominantly using cooking oil while cooking	Groundnut oil	44 (37.3%)
	Palm oil	25 (21.2%)
	Refined	4 (3.4%)
	Sunflower	45 (38.1%)
Physical activity***	Heavy	14 (11.9%)
	Moderate	98 (83.1%)
	Sedentary	6 (5.1%)

**Figure 1:**
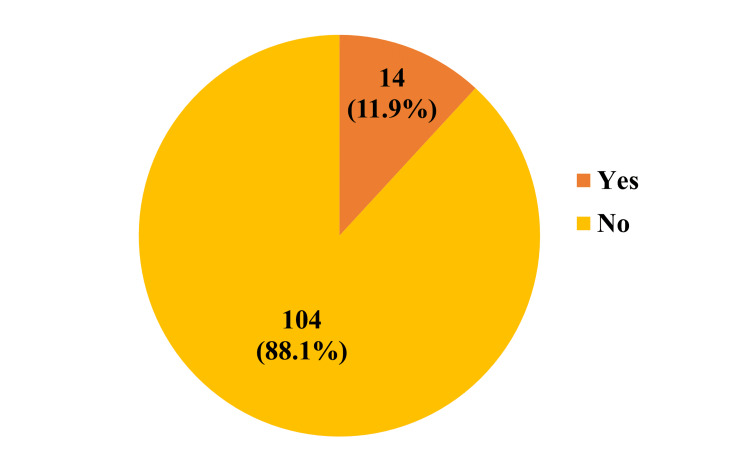
Prevalence of T2DM among professional drivers T2DM: type 2 diabetes mellitus

The prevalence of diabetes among professional drivers in the study was 14 (11.9%). The prevalence of diabetes is shown in Figure 1.

**Figure 2:**
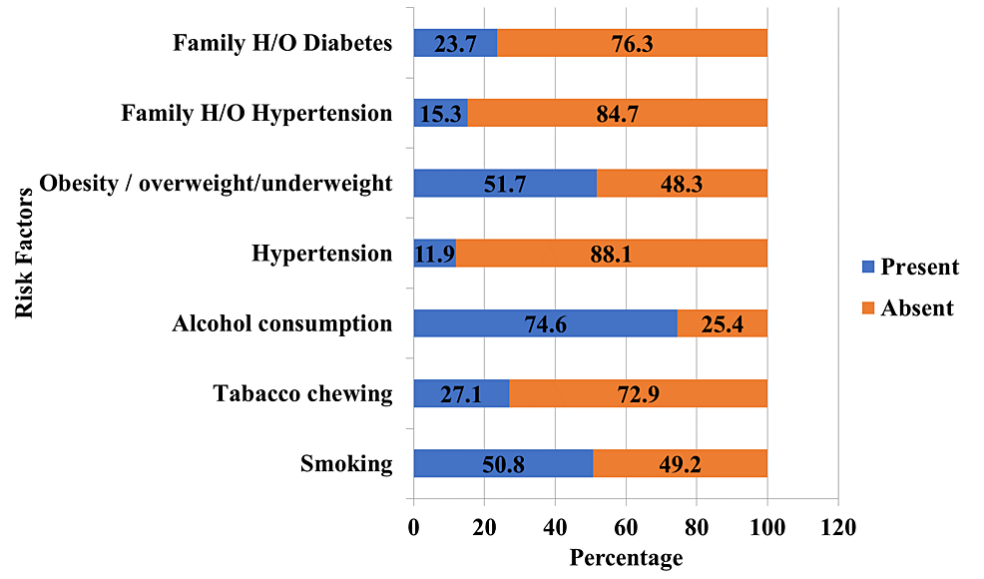
Prevelance of risk factors for T2DM among professional drivers T2DM: type 2 diabetes mellitus

The prevalence of risk factors for T2DM is given in Figure 2, where 50.8% of the participants smoked, 27.1% chewed tobacco, 74.6% consumed alcohol, 11.9% had hypertension, 51.7% were obese, overweight, or underweight, and 15.3% had a family history of hypertension.

The association between sociodemographic factors and T2DM among professional drivers is shown in Table 6. Participants aged 21-35 years and >65 years did not develop T2DM unlike the age groups of 36-50 (5.7%) and 51-65 years (27.3%). Likewise, most of the participants who studied up to secondary education (66.7%) developed T2DM followed by those who had primary education (15.4%) or were illiterate (10.4%) while those who studied up to higher secondary or graduated did not have T2DM. Such differences in age group (p-0.001) and education (p<0.001) were statistically significant. More participants who belonged to a nuclear family (14.3%) and to class 2 socioeconomic class (21.1%) developed T2DM when compared to their counterparts but this was not statistically significant (p > 0.05).

**Table 6:** Association between T2DM and general characteristics in professional drivers. Chi-square test was applied *p value <0.05 and it is statistically significant T2DM: type 2 diabetes mellitus

General characteristics		Type 2 Diabetes Mellitus		p-value
		Yes	No	
Age	21-35	0	33 (100%)	0.001^*^
	36-50	2 (5.7%)	33 (94.3%)	
	51-65	12 (27.3%)	32 (72.7%)	
	> 65	0	6 (100%)	
Education	Degree	0	18 (100%)	<0.001^*^
	Higher Secondary	0	4 (100%)	
	Secondary education	4 (66.7%)	2 (33.3%)	
	Primary education	2 (15.4%)	11 (84.6%)	
	No education	8 (10.4%)	69 (89.6%)	
Type of Family	Joint	0	6 (100%)	0.198
	Nuclear	14 (14.3%)	84 (85.7%)	
	Three generations	0	14 (100%)	
Socio- economic Status	Class 1	0	18 (100%)	0.103
	Class 2	8 (21.1%)	30 (78.9%)	
	Class 3	4 (12.1%)	29 (87.9%)	
	Class 4	2 (6.9%)	27 (93.1%)	

Table 7 shows the association between risk factors and diabetes among professional drivers. A higher percentage of ex-tobacco chewing (100%), obese (35.3%), hypertensive (63.6%), ex-smoker (60%), and central obesity (21.1%) have developed T2DM when compared to their counterparts. Such differences were statistically significant (p<0.05). There was a significant association between T2DM and smoking, tobacco chewing, hypertension, elevated BMI, and elevated waist circumference.

**Table 7:** Association between T2DM and risk factors in professional drivers. Chi-square test was used * p value <0.05 and it is statistically significant; # Fischer's exact test was used T2DM: type 2 diabetes mellitus

Risk Factors		Type 2 Diabetes Mellitus		p-value
		Yes	No	
Smoking	Current	2 (4.5%)	42 (95.5%)	0.002*
	Ex user	6 (60%)	10 (40%)	
	Non-user	6 (10.3%)	52 (89.7%)	
Tobacco chewing	Current	2 (6.7%)	28 (93.3%)	0.000*
	Ex user	2 (100%)	0	
	Non-user	10 (11.6%)	76 (88.4%)	
Alcohol consumption	Current	8 (11.8%)	60 (88.2%)	0.360
	Ex user	4 (20%)	16 (80%)	
	Non-user	2 (6.7%)	28 (93.3%)	
Diet intake#	Mixed diet	12 (10.9%)	98 (89.1%)	0.241
	Vegetarian	2 (25%)	6 (75%)	
Salt intake#	Yes	2 (5.9%)	32 (94.1%)	0.345
	No	12 (14.3%)	72 (85.7%)	
Physical activity	Heavy	0	14 (100%)	0.198
	Moderate	14 (14.3%)	84 (85.7%)	
	Sedentary	0	6 (100%)	
Hypertension#	Yes	8 (57.1%)	6 (42.9%)	<0.001*
	No	6 (5.8%)	98 (94.2%)	
Hypertension-classification	Hypertensive	14 (63.6%)	8 (36.4%)	<0.001*
	Normotensive	0	51 (100%)	
	Pre-Hypertensive	0	45 (100%)	
Obesity-BMI index	Underweight	0 (0%)	4 (100%)	0.012*
	Normal	4 (7%)	53 (95%)	
	Overweight	4 (10%)	36 (90%)	
	Obese	6 (35.3%)	11 (64.7%)	
Waist circumference#	Central obesity	12 (21.1%)	45 (78.9%)	0.004*
	Normal	2 (3.3%)	59(96.7%)	

## Discussion

In our study, the prevalence of diabetes was 14 (11.9%) amongst the professional male drivers in Perambalur Municipality. A study done in South Karnataka showed a similar prevalence of diabetes (11.1%) among drivers [10] along with a study in three regions (East/West/South) of India, which showed a prevalence of 11-18% [19]. In contrast, in a study conducted by Yosef in Ethiopia among truck drivers (2018), 32 (8%) had diabetes mellitus [2], and a study in Iran also showed a 9.1% prevalence of diabetes [20], which is lower than the current study. Another study by Sangaleti et al. in Brazil states the prevalence of diabetes among truck drivers was 16.4% [21]. The variation observed compared to other studies could be owing to the differences in method, sample size, and operational definitions used. Besides, the socioeconomic, behavioral/lifestyle, and cultural and educational profiles may create a significant variation.

In our study, the risk factors contributing to the development of T2DM among drivers were age, education, smoking, tobacco chewing, hypertension, and obesity. Similarly, a study with Polish drivers by Marcinkiewiz et al. states that increasing age plays an important role in the development of diabetes [1]. Budreviciute et al.'s study showed that harmful habits, such as smoking and drinking alcohol, which were gained by adolescent young people, can significantly contribute to NCD risk [22]. The unhealthy habits may continue during adulthood, which influences the progress of NCDs. So, age plays a major role in the development of diabetes among drivers.

In our study, participants involved in alcohol consumption, smoking, and tobacco chewing were 68 (57.6%), 44 (37.3%), and 30 (25.4%), respectively. Smoking and tobacco chewing were associated with T2DM among drivers (p = 0.002; statistically significant). Similarly, a study conducted by Jaganmohan et al. in Nellore showed an association between smoking and diabetes among drivers (p = 0.003) [23]. A study conducted in Hyderabad showed that 44.07% were chronic smokers, 47.46% used chewable tobacco products, and 57.63% were found to be addicted to alcohol, which were associated with the development of diabetes mellitus [11].

In our study, the prevalence of hypertension among the drivers was 14 (11.9%). In contrast, in studies conducted in Hyderabad [11], South Karnataka [10], Iran [6], and Poland [1], the prevalence of hypertension among long-distance truck drivers was 45.76%, 28.9%, 42.9%, and 36.7%, respectively.

Nearly 14.4% of the drivers were obese, while 33.9% were pre-obese, and central obesity was observed among 48.3% of the participants. In comparison with the current study of 14.4% obesity, a survey conducted in South Karnataka [10], Iran [6], and Poland [1] among long-distance truck drivers showed a higher prevalence of obesity of 40%, 23%, and 17.4%, respectively. The longer duration of driving hours creates more hours of sitting while driving resulting in overweight and obesity.

Limitations

A causal link between risk variables and T2DM could not be drawn because of the study’s cross-sectional design. We could not infer the risk factors for the development of T2DM among drivers because of the limited sample size and non-probability technique. Based only on their medical history, we assumed that professional drivers had T2DM, which may have resulted in social desirability bias and recollection bias. Additionally, we did not measure fasting blood sugar (FBS), postprandial blood sugar (PPBS), or glycated hemoglobin (HbA1C). Specifically, qualitative data collection and analysis may prove useful in providing insight into how and why various forms of risk factors may influence the development of diabetes among professional drivers differently. Finally, these findings are specific to professional drivers working in Perambalur, India, and should not be generalized.

## Conclusions

The prevalence of T2DM was 11.9%. The major risk factors for the development of T2DM among professional drivers were age, education, excessive body weight, high blood pressure, and personal habits like smoking and tobacco chewing. We should conduct similar studies on a larger scale at multiple centers and involve more refined techniques and expertise to yield better results.

The risk factors for T2DM show a need to undertake multidimensional actions that target specific professions and involve various healthcare sectors. To ascertain the prevalence and severity of T2DM, the transportation departments should consider doing pre-placement examinations with the assistance of physicians. These findings further substantiate the need for preventive and health-promotive interventions like the encouragement of regular physical activity and quitting harmful habits like tobacco and alcohol to combat the rising risk factors for T2DM among drivers.
